# 32 Effect of Oral Ketamine on Analgesia During Burn Wound Care

**DOI:** 10.1093/jbcr/irac012.035

**Published:** 2022-03-23

**Authors:** Jill Rozynski, Charlotte Larger, Alicia Patton, Amber Kohler, Arek J Wiktor

**Affiliations:** University of Colorado ABA Verified Burn and Frostbite Center, Denver, Colorado; University of Colorado Hospital ABA Verified Burn and Frostbite Center, Denver, Colorado; University of Colorado Hospital, Denver, Colorado; University of Colorado School of Medicine, Denver, Colorado; University of Colorado Burn Center, Aurora, Colorado

## Abstract

**Introduction:**

Pain and anxiety can be difficult to control in the burn patient. Ketamine, a NMDA receptor antagonist can be utilized, however intravenous (IV) dosing requires increased monitoring. In contrast, oral ketamine (O.K.) is reported to have fewer side effects, and can be administered without a higher level of care. Little is known about dosing and efficacy of O.K. in the treatment of burn patients. We sought to examine the use of O.K. in our burn population.

**Methods:**

This was a cross-sectional comparative quality improvement study conducted at our ABA verified burn center from January 2021through September 2021. Inclusion criteria were in patients experiencing refractory pain and anxiety after receiving fentanyl and midazolam during wound therapy one day prior to first O.K. administration, along with completion of pre-post patient assessment surveys. Patients were administered 50mg-150mg of ketamine in an oral suspension mixed with 30mL-120mL juice. Effectiveness of the O.K. was measured using pre-post patient surveys, pre-post IV pain medication usage, and subjective staff evaluation of pre-post tolerance of debridement. Baseline demographics were recorded, along with adverse events. O.K. was deemed effective by improved survey scores, improved tolerance to debridement, and reduction in IV medication requirements. Students T-test was performed to determine significance.

**Results:**

A total of 71 patients were given O.K, and 32 met inclusion criteria. Baseline demographics included: 19 male (59%), median age 36 years (range14-66), median TBSA 9.9% (range 1.6%-33.2%). O.K. reduced mean fentanyl use by 33% (pre 199.2mcg- post 123.5mcg, p< 0.01) and mean midazolam use by 39% (pre 1.4mg - post 0.7mg, p< 0.01). On a scale of 1-10 (1 best, 10 worst) mean pain scores improved 38.8% (pre 8 – post 4.6, p< 0.01), anxiety by 36.6% (pre 6.5 – post 3.5, p< 0.01), and overall experience by 37.5% (pre 5.9 – post 3, p< 0.01). Mean O.K. effective dose was 50mg. Staff noted O.K. improved the patient’s ability to tolerate debridement; uncooperative and inconsolable patients participated in their own wound care, and reported to prefer tub time with O.K. One patient experienced psychotomimetic effects, while one patient requested discontinuation due to increased anxiety 4 hours after O.K. administration.

**Conclusions:**

O.K. appears to be efficacious in improving pain and anxiety during wound care while being well tolerated by patients. It also subjectively improved the wound care therapy experience.

